# Pimavanserin Treatment for Parkinson's Disease Psychosis in Clinical Practice

**DOI:** 10.1155/2021/2603641

**Published:** 2021-01-04

**Authors:** Khashayar Dashtipour, Fiona Gupta, Robert A. Hauser, Cherian A. Karunapuzha, John C. Morgan

**Affiliations:** ^1^Loma Linda University School of Medicine, 11370 Anderson Street, Loma Linda, CA 92354, USA; ^2^Mount Sinai Hospital, 1468 Madison Avenue, New York, NY 10029, USA; ^3^University of South Florida College of Medicine, 4001 East Fletcher Avenue, Tampa, FL 33160, USA; ^4^The Meinders Neuroscience Institute, 4120 W Memorial Rd, Oklahoma, OK 73120, USA; ^5^Medical College of Georgia, Augusta University, 1120 15th Street, Augusta, GA 30912, USA

## Abstract

**Background:**

Parkinson's disease psychosis (PDP) is a common, nonmotor symptom of Parkinson's disease (PD), which may affect up to 60% of patients and is associated with impaired quality of life, increased healthcare costs, and nursing home placement, among other adverse outcomes. Characteristic symptoms of PDP include illusions; visual, auditory, tactile, and olfactory hallucinations; and delusions. PDP symptoms typically progress over its course from being mild, infrequent, and often untroubling to complex, sometimes constant, and potentially highly disturbing. PDP has traditionally been treated with atypical antipsychotics (e.g., clozapine and quetiapine) although these are not approved for this indication and clozapine requires frequent white blood cell count monitoring due to the risk of agranulocytosis. Pimavanserin is a newer atypical antipsychotic with highly selective binding to serotonergic receptors, no evidence for worsening motor symptoms in PD, and no need for white blood cell count monitoring. It is currently the only approved medication indicated for PDP treatment. However, because it was approved relatively recently (2016), clinical experience with pimavanserin is limited. *Case Presentations*. A wide variety of representative clinical scenarios are presented, each with distinct variables and complications. Issues addressed include distinguishing PDP from similar symptoms caused by other disorders such as dementia, coordinating pimavanserin with other PD medications and with deep brain stimulation, adapting pimavanserin dosing for optimal benefit and tolerability, and recognizing variability of PDP symptoms due to patients' changing life circumstances.

**Conclusions:**

These scenarios provide multiple insights regarding PDP management and the role of pimavanserin. Effective treatment of PDP may reduce disturbing symptoms of psychosis, thus improving patient function and quality of life. In addition, effective pharmacotherapy for PDP may also facilitate the use of other medications needed to treat neurological symptoms of PD (e.g., tremor, bradykinesia, and dyskinesia), although they may also have adverse effects that contribute to symptoms of PDP.

## 1. Introduction

Parkinson's disease psychosis (PDP) is a nonmotor symptom characterized by illusions, false sense of presence, and/or hallucinations or delusions that are recurrent or continuous for at least a month and are not attributable to another etiology [1]. PDP exists on a spectrum from mild and untroubling to severe and disabling. A progressive condition, it is one of the leading causes of nursing home placement for patients with Parkinson's disease (PD) [2–4]. Although PDP is common, it is frequently underrecognized or undertreated by physicians [5]. The care of patients with PDP represents a significant healthcare financial burden; in a Medicare survey of claims data from 2000 to 2010, patients with PDP were among the groups with the highest resource utilization [6]. In addition to healthcare costs, PDP markedly impacts the quality of life of patients and their caregivers [7, 8].

Multiple studies have reported that one-fourth to one-third of patients with PD experience PDP [1, 3, 9–12]. However, the severity of the symptoms varies and can range from minor and easily recognized, such as illusions (misperceptions of real stimuli such as a tree branch mistaken for a cat) or passage hallucinations (fleeting hallucinations of objects passing in the peripheral vision), wherein patients have insight into their psychoses, to severe and frightening, such as complex visual hallucinations or delusions without insight [4]. Longitudinal study data show that benign hallucinations often progress to more serious symptoms, and eventually, up to 60% of patients with PD may develop PDP [13, 14]. Factors that may contribute to the development of PDP include older age and older age at onset of disease, axial parkinsonism, higher doses of dopaminergic medication, longer disease duration, more advanced disease, rapid eye movement sleep behavior disorder (RBD), impaired visual acuity, a family history of dementia, or the presence of cognitive impairment or dementia [13]. The mean time to onset of hallucinations was 10.7 years, as recorded in the Sydney multicenter study of PD patients [15].

Visual hallucinations are the most common presentation of PDP, but other forms of hallucinations may occur, such as auditory, tactile, and olfactory [4]. Delusions are often characterized by paranoia, jealousy, or persecution and usually occur with the progression of illusions and hallucinations [1, 4]. However, a literature review found that delusions in PDP commonly manifest in isolation [16]. Delusions have been reported in approximately 5% to 25% of patients with PDP [1, 3, 17, 18].

The first step in assessing the patient with PDP is to identify possible reversible etiologies, such as infection or toxic metabolic states (e.g., renal or hepatic failure). Diagnoses such as encephalitis and neurosyphilis can present in afflicted individuals as bizarre behavior or cognitive changes. Patients should be assessed for any current medication or illicit drug use that may be contributing to symptoms of psychosis, and potentially culpable substances should be evaluated for discontinuation [19]. Consider gradually reducing or eliminating some antiparkinsonian medications so as to reduce hallucinations but without seriously impacting their ability to treat parkinsonism. Anticholinergics, amantadine, MAO-B inhibitors, dopamine agonists, and levodopa/carbidopa can all produce hallucination side effects to varying degrees [20–22]. Clinicians should also be made aware of any recent traumatic life events, as that is a strong risk factor in the development of psychosis [23]. If there is no known non-PD etiology and psychosis continues, antipsychotics are usually first-line therapy. Antipsychotics are indicated for psychiatric disorders such as schizophrenia and bipolar disorder, but also are commonly used off-label for the management of PDP. Among the antipsychotics, only clozapine and quetiapine have been widely prescribed for the treatment of PDP [24, 25]. However, many atypical antipsychotics may cause worsening of the motor function in PD patients because of dopamine receptor blockade, and they increase the risks of cardiometabolic adverse effects (AEs) including weight gain, diabetes, dyslipidemia, and hypertension [26, 27]. The use of clozapine requires routine laboratory blood monitoring for the risk of agranulocytosis [24]. Quetiapine does not require laboratory monitoring but has not demonstrated better efficacy than placebo for PDP and is associated with cardiometabolic AEs [25, 28–32]. In the setting of PD dementia, cholinesterase inhibitors (rivastigmine, donepezil, and galantamine) may be useful in reducing psychoses [33, 34].

Pimavanserin is a nondopaminergic, selective 5-HT_2A_ inverse agonist/antagonist that was approved by the US Food and Drug Administration (on April 29, 2016) for the treatment of hallucinations and delusions associated with PDP [35]. Pimavanserin binds specifically to 5-HT_2a_ receptors and, to a lesser extent, to 5-HT_2c_ receptors but has no binding affinity for dopaminergic, adrenergic, muscarinic, or histaminergic receptors; thus, it does not worsen the motor function in PD unlike most other antipsychotics [36, 37]. It is currently the only medication specifically indicated for PDP. Like other antipsychotics, pimavanserin has a boxed warning regarding increased mortality in elderly patients with dementia-related psychosis, stating that pimavanserin is not approved for the treatment of patients with dementia-related psychosis unrelated to the hallucinations and delusions associated with PDP. The safety and efficacy of pimavanserin 34 mg for the treatment of PDP were demonstrated in a pivotal phase 3, randomized, double-blind, placebo-controlled clinical trial [38]. Evidence-based reviews by expert panels of the Movement Disorder Society and the American Geriatrics Society have recommended the use of pimavanserin for the treatment of PDP symptoms [36, 39].

Because it was approved only recently, however, neurologists, psychiatrists, and other healthcare professionals lack adequate clinical experience with pimavanserin. In this paper, we present several case studies contributed by the authors to illustrate a variety of clinical scenarios and lessons learned from these experiences where pimavanserin was prescribed for managing PDP symptoms.

## 2. Methods

Patients who had been prescribed pimavanserin for treating hallucinations and delusions associated with PDP were identified from a review of the medical records of neurology practices at five medical centers in the United States. Cases were identified as patients with PD who had an illustrative medical history or symptoms or had experienced an inadequate response to previous treatment regimens. All cases were reviewed by the author panel for suitability and interest at an in-person round table discussion. The initial visit for patients included in this case series occurred between 2016 and 2018. Data were extracted from medical records by the authors or an experienced healthcare professional in their clinic, and case conclusions are the product of the author panel as a whole. The confidentiality of patient identity was maintained. The American Academy of Neurology (AAN) considers the classification of evidence as Class IV and classification of recommendation as U for a case series.

## 3. Case Series Presentation For case series presentation summaries, see Tables 1 and 2

### 3.1. Case Study 1

A 79-year-old right-handed man with PD diagnosed in 2013 was referred for management of PD symptoms. At the time of diagnosis, he was treated with rasagiline, but because of progression of symptoms (increased slowness of movements and tremor), he was switched to carbidopa/levodopa (CD/LD) 25/100 three times a day. At the first visit, in the summer of 2016, his main complaint was tremor in the morning and sometimes late afternoon. His wife noticed that he could not handle complex tasks anymore and had stopped engaging in conversations. She also noticed changes in behavior such as constant questioning about their bank statement, and she thought he was developing dementia. The patient had a history of mild depression and walked slowly at times but had no history of falls. On examination, he was alert and oriented to person and location but got the day's date wrong, had limited knowledge, and was quiet during the examination as his wife gave most of the history. He had taken CD/LD 90 minutes before the exam and showed mild intermittent left-hand tremor with mild bradykinesia on the right side with normal tone and gait. The assessment was PD with mild motor fluctuation, and rasagiline 1 mg was added to his CD/LD regimen.

Two months later at the follow-up visit, the patient showed significant improvement of motor symptoms; however, his wife was still concerned about symptoms of possible dementia. He was evaluated for dementia, and he scored 27/30 on the Mini-Mental State Exam. He did not know what day it was, made 1 error on spelling “world” backward, and missed 1 word out of 3 for recall. His wife clarified that the patient was never previously concerned about their finances because she managed them. However, she stated that, in the past year, the patient's behavior had changed dramatically in that he continually asked to check their bank statements every month, inquiring where the money was going as though suddenly mistrustful of her, and became angry if she did not show him the statements.

In the assessment, the patient's behavior was diagnosed as PDP with associated delusion (patient being suspicious of his wife), and pimavanserin 34 mg/d was started. One month later, at a follow-up visit, the patient's wife stated that the “dementia medication” (pimavanserin) was very helpful. Many people at church said her husband had changed, was more talkative, and engaged more in conversation. In addition, he had stopped asking to review the bank statements. During evaluation and examination, the patient participated more in history-taking than in previous visits (the Mini-Mental State Exam was not repeated). No AEs were noted.

### 3.2. Case Study 2

A 71-year-old man with a history of diabetes, hypertension, and tremor-predominant PD since 2004 was seen for the first time in 2011 for the chief complaint of generalized dyskinesias and tremor. His motor symptoms were under reasonable control with bilateral subthalamic nucleus deep brain stimulation (DBS) and CD/LD 25/250 mg three times daily. He also took citalopram 20 mg/d for depression. Since 2016, he had begun to show a mild cognitive decline with attention and concentration deficits, mood swings, and excessive anger. Concurrently, the patient began having intermittent passage phenomena consisting of fleeting shadows. The hallucinations gradually became well-formed and included little children and dwarves, which appeared funny to him. Occasionally, the patient would lose insight, and his wife would observe him interacting with the hallucinations. The PDP was initially treated with quetiapine 12.5 mg/d and increased after 1 week to 25 mg/d. Although quetiapine made the patient sleepy, his hallucinations were partially controlled. However, he underwent knee surgery during this time, and when recovering postanesthesia, he experienced worsened hallucinations and delirium. To counter these symptoms, quetiapine was increased to 50 mg/d and then to 25 mg in the morning and 50 mg in the evening; this dosage resulted in excessive daytime sedation and an increase in “foggy” thinking, without full control of hallucinations. However, the patient's delirium gradually improved on this dosage after a week in the hospital, and he was discharged.

Following discharge, pimavanserin was initiated in August 2017 at 17 mg/d for 2 weeks while quetiapine was simultaneously tapered off by 25 mg per week. After the second week of pimavanserin 17 mg/d, quetiapine was discontinued, and pimavanserin was increased to 34 mg/d; however, this dose led to all-day sedation, and the pimavanserin dose was reduced back to 17 mg/d. After 5 weeks of pimavanserin treatment, the PDP symptoms were controlled.

### 3.3. Case Study 3

A 79-year-old retired male college professor, with a well-established diagnosis of PD since 2011, was seen in the clinic in 2014 for complaints of auditory hallucinations. His history included motor fluctuations with wearing off at 3.5–4 hours, early-morning akinesia, and painful right foot dystonia. On examination, he showed rest tremor, bradykinesia, and right-sided rigidity. In February 2017, the patient began experiencing passage phenomena including vague shadows at the edge of his vision, birds continuously chirping softly in a musical tone, and faint intermittent music. He had partial hearing loss in both ears for years preceding these PDP symptoms, and he had not reported any delusions previously. In mid-2017, the auditory hallucinations were intrusive enough that he volunteered the symptoms at a routine screening for psychosis and sought treatment.

With no previous antipsychotic treatment, the patient was started on pimavanserin 34 mg/d. He experienced sedation, and after 5 days, the dose was reduced to 17 mg. The sedation persisted, however, starting approximately 6-7 hours after taking the medication. The time of dosing was shifted to early evening, whereupon the patient no longer experienced daytime sedation. PDP symptoms (auditory hallucinations) resolved completely after approximately 4 weeks of pimavanserin 17 mg/d, and treatment was continued at this dose.

### 3.4. Case Study 4

A 71-year-old male retired stockbroker, with well-established, tremor-dominant PD with motor onset in 2006, was referred to the movement disorders clinic in 2018. Motor symptoms were responsive to CD/LD 25/100 mg one and one-half tablets three times daily and rasagiline 1 mg/d. He was also taking sertraline 100 mg/d for depression. His history revealed wearing-off symptoms after 4-5 hours with no dyskinesia. Before the consulting visit, the patient reported persistent daily visual hallucinations in the evening with vague shadows of people walking beside him. He had severe nightmares in 2016 after amantadine was initiated for tremor, and these symptoms stopped when amantadine was discontinued. In early 2017, the patient also reported experiencing passage hallucinations intermittently in the evening similar to those experienced earlier, although he did not report this to his physician at the time. By 2018, when the patient was seen at the clinic, he was experiencing persistent false perceptions of people behind him and almost daily hallucinations of insects and animals crawling under his sheet while he was trying to sleep, as well as intermittent and vivid images of exotic animals, like a black panther in his living room. He also developed severe nightmares and woke up crying. Although these spells were very intrusive, his referring physician had not identified or treated them.

Following evaluation, the patient was started on pimavanserin 34 mg/d and after 3 weeks showed a reduction in PDP symptoms. Complete resolution of symptoms, including severe nightmares and potential RBD symptoms, was reported after about 6 weeks. Of note, CD/LD 25/100 mg was increased to 2 tablets three times daily concomitant with pimavanserin, without worsening of PDP symptoms.

### 3.5. Case Study 5

A 68-year-old left-handed man with PD for 10 years was first seen in July 2016 with the chief complaint of difficulty walking. Consultation with the patient and his wife revealed motor fluctuations with difficulty walking almost 30 minutes before each dose of CD/LD. The wife also mentioned some short-term memory loss, but no mood changes or hallucinations. Examination during on-time showed mild bradykinesia on the right side with mildly increased tone and intermittent, mild resting tremor. The patient was able to stand up, cross arms, and walk with normal posture, stride, and balance, with less arm swing on the right side. During off-time, examination showed moderate to severe bradykinesia on the right side and mild to moderate bradykinesia on the left; increased tone on the right side and mild to moderate on the left; and constant tremor. The patient needed to push himself off the chair and showed mild freezing at gait initiation.

Following examination, the patient was assessed as having PD with motor fluctuations, and selegiline 5 mg twice a day was added to his regimen. After 6 weeks, the patient noticed improvement of motor fluctuation and felt stronger during on-time for the first 4 weeks. However, he stopped the selegiline after 2 weeks when he noted that his hallucinations had not stopped. Following discontinuation of selegiline, the hallucinations stopped, but the patient still complained of motor fluctuations. At the follow-up visit, selegiline was restarted and pimavanserin 34 mg/d was added for recurrence of hallucinations. The patient tolerated selegiline in combination with pimavanserin 34 mg/d, had a significant reduction of off-time, and felt better overall during on-time. At a 6-week follow-up visit, the patient had continued taking selegiline and pimavanserin with no hallucinations.

### 3.6. Case Study 6

An 84-year-old woman with PD for 20 years, taking CD/LD 25/100 mg qid and pramipexole 1.5 mg three times daily, was seen at the movement disorders clinic. She presented with minimal persisting tremor over several years with increasing bradykinesia, dyskinesia of the head, rigidity, and gait freezing over time. Symptoms were worse on the right side. Her history revealed continued wearing off of dopaminergic therapies with head, truncal, and appendicular dyskinesias. Trials of amantadine and rasagiline had failed because of AEs including hallucinations. Neuropsychological testing done 4 years before the initial visit had demonstrated PD-associated mild cognitive impairment.

Fifteen months before the initial visit, she experienced an apparent visual hallucination, seeing a skunk near a garbage can that her husband did not see at the same time. At the time of the initial visit, the patient's hallucinations mainly involved her children sitting around the house—in her bedroom, living room, and elsewhere—in a nonthreatening way. She also reported seeing animals that others did not see. A reduction in pramipexole dose was attempted to control her hallucinations, but this was unsuccessful because of worsening of motor symptoms and freezing of gait. Over time, the hallucinations evolved from being occasional to constant throughout the day and evening. Initially, the patient understood the hallucinations were not real, but insight deteriorated such that she was increasingly questioning her husband whether they were real. No infection, fever, or significant intercurrent illnesses were present to cause delirium as a differential diagnosis of the phenomena.

The patient was started on pimavanserin 34 mg/d, and after several weeks of treatment, her visual hallucinations of her children decreased to a rate of several times a week. In addition, the skunk she saw by the garbage can did not reappear.

### 3.7. Case Study 7

A 73-year-old man with PD diagnosed in 2016 was evaluated at the neurology clinic in November 2017. PD symptoms had begun in 2011 with rest tremor in the left fifth finger. Initially, the patient was treated successfully with CD/LD 25/100 three times daily in 2016. The patient had a history of moderate cognitive dysfunction and difficulty with some tasks of daily living (e.g., calculating tips and getting around a new city). He also had a history of depression and anxiety, ongoing at the time of evaluation; he had been taking duloxetine 60 mg twice a day for several years at the time of evaluation. On examination, the patient showed slight bilateral resting tremor; mild to moderate bilateral reduced fine movements; tone mildly increased on the right and normal on the left; gait with normal strides; and arm swing moderately reduced bilaterally. The patient's spouse reported additional symptoms consistent with RBD, with frequent motor episodes at night. The patient also reported frequent awakenings at night, often accompanied by hallucinations, which typically consisted of furry animals at the foot of the bed. The following management was planned as sequential steps over a 7-day period: (1) reduce and discontinue duloxetine and start escitalopram oxalate 10 mg/d in the morning; (2) add pimavanserin 34 mg/d at bedtime; and (3) add donepezil 10 mg/d in the morning.

Three months later at the follow-up visit, the patient reported that his hallucinations had resolved within 1-2 weeks of starting pimavanserin, that vivid dreams (nocturnal awakenings) and continuity of sleep were much improved, and that cognition had improved slightly (presumably attributable to donepezil). However, RBD symptoms persisted; therefore, clonazepam 0.5 mg at bedtime was prescribed. The patient reported low mood, so he was switched from escitalopram oxalate 10 mg/d in the morning to venlafaxine extended-release 75 mg/d in the morning.

### 3.8. Case Study 8

A 68-year-old right-handed man with a 9-year history of PD and taking levodopa was first seen to evaluate as a candidate to receive DBS. He presented with severe refractory tremors and motor fluctuations. Preoperative evaluation indicated that the patient would be a candidate for DBS, and neuropsychological testing indicated no contraindications. The patient underwent bilateral subthalamic nucleus DBS and showed significant motor improvement, particularly for refractory tremor. Consequently, the levodopa dose was reduced, and at 4 to 6 weeks following the DBS, the patient had developed cognitive decline, verbal dysfluency, auditory hallucinations (e.g., hearing a door slam), agitation, and paranoia. He also exhibited delusions and paranoia, suspecting his wife of having an affair and following her around the house. The DBS was discontinued but with no change in mental or psychiatric symptoms and with profound worsening of motor symptoms. Therefore, pimavanserin 34 mg/d was started, and by 4 weeks, the patient reported fewer hallucinations, less paranoia, and better ability to stay home independently. The patient's speech impairment persisted, and currently, he is undergoing cognitive behavioral therapy.

### 3.9. Case Study 9

A 69-year-old woman with an established diagnosis of PD since 2002 and taking levodopa was seen in 2017. Symptoms and complaints consisted of tremors, bradykinesia, rigidity, and severe dyskinesia, with a history of longstanding depression and mild cognitive impairment. She underwent bilateral globus pallidus internus DBS in 2015. Her medications included amantadine and rotigotine. She reported experiencing passage hallucinations consisting of fleeting shadows in 2014. By 2017, she reported feeling a presence in dark corners (e.g., a demon) while going to sleep at night but never reported actually seeing anything. Over the ensuing months, the patient grew afraid of who might come through the front door of her house; becoming paranoid about home invasion, she would not open the door when anyone rang the bell. The patient was transferred to the clinic.

Initial management for PDP symptoms was to discontinue amantadine and then rotigotine, which resulted in the patient becoming slower in movement and more tremulous. Pimavanserin 34 mg/d was initiated. The levodopa dose was not increased for fear of worsening hallucinations. After 4 weeks of this treatment, and with a reduction of the levodopa dose, PDP symptoms were only partly resolved. Later, the patient was admitted to a psychiatric unit for further medical management, where quetiapine was added and quickly titrated to 150 mg/d over a week. Quetiapine along with continued pimavanserin 34 mg/d calmed the patient at night so that she would not wake up terrified.

### 3.10. Case Study 10

A 74-year-old right-handed woman with a 12-year history of PD was seen at the movement disorders clinic with chief complaints of left upper-extremity tremor, decreased dexterity, and gait instability. The history revealed that she had responded well to levodopa initially, but within 2 years of treatment, she developed mild to moderate motor fluctuations. She had more than a 20-year history of depression with anxiety and a history of electroconvulsive therapy. The patient had developed visual hallucinations during a period of stress and depression (stressors included a daughter who wanted to move her to assisted living). The hallucinations included a rock that seemed to turn into a monster and animals in the house.

Initially, nonpharmacological approaches were discussed, including reducing stress in her life, speaking with her children to resolve differences and allay fears, and modifying her home environment with improvements such as better lighting to help ward off misperceptions (illusions). Four months after this consultation, the patient was hospitalized for an infection and had a prolonged rehabilitation stay. During this time, the patient developed worsening hallucinations, agitation, and delusions, such as believing that her roommate was “out to get her.” The patient was then started on quetiapine at a dose of 100 mg at discharge from the rehab center. At a subsequent follow-up visit at the clinic, pimavanserin 34 mg/d was initiated along with quetiapine to improve symptom control. At 6 weeks after starting pimavanserin, the patient reported significant reductions in hallucinations, depression, and anxiety. Quetiapine was tapered from 100 mg/d to 50 mg/d, but the patient developed worsening anxiety and sleep disturbances. The patient currently is taking both pimavanserin 34 mg/d and quetiapine 75 mg/d at bedtime.

### 3.11. Case Study 11

A 76-year-old man with a 4-year history of PD motor symptoms was seen at an initial visit with a chief complaint of shuffling gait, slowness, and drooling. On examination, he showed cognitive impairment and akinetic-rigid parkinsonism with mild bilateral resting tremor without dyskinesias. He had developed cognitive impairment over time. Three years before this visit (1 year after the onset of PD symptoms), the patient had also developed visual hallucinations, such as seeing groups of people outside his room in an assisted living facility, heads of people, a fluffy cat that no one else saw, and a woman standing in a garbage can with a man wearing a fancy hat. He also developed auditory hallucinations consisting of a person who would knock at his door and talk about an event that had not occurred. Hallucinations occurred daily and were bothersome.

The patient was started on pimavanserin 34 mg/d. Within 4–6 weeks, he experienced marked improvement of visual hallucinations, which decreased in frequency to only rare occurrences that were not bothersome, and cessation of auditory hallucinations. No AEs were reported after 9 months of therapy.

## 4. Discussion and Conclusion

This case series describes managing PDP using pimavanserin in patients treated in the clinical practice setting. These cases portray several issues that may occur in practice, such as coadministration of pimavanserin with other antipsychotics such as quetiapine, medication AEs, the effects of other comorbidities such as cognitive impairment, and potential dose intolerance with 34 mg of pimavanserin. Additionally, these cases demonstrate the risk of PDP in DBS patients and the application of pimavanserin in this setting.

These clinical vignettes emphasize the importance of obtaining a careful history to recognize PDP symptoms and interpreting patients' descriptions of their complaints and symptoms. One case study demonstrated RBD as a common comorbidity in PD that is associated with hallucinations and must be differentiated from PDP with a clear and detailed history [40]. Dreams must also be differentiated from hallucinations. Another case example demonstrated that when evaluating PD patients whose behaviors resemble dementia, clinicians should consider the possibility of PDP, especially when the patient demonstrates suspicions, paranoia, and personality changes. This consideration, in turn, raises the question of whether such symptoms should be aggressively treated as PDP (i.e., with pimavanserin or other antipsychotic therapy), given the associated risks that PDP may worsen patients' quality of life and increase the likelihood of nursing home placement [3, 7]. These cases also demonstrate that pimavanserin can be added to PD medications without compromising its efficacy and tolerability. In fact, in two cases, after controlling PDP symptoms with pimavanserin, clinicians were able to increase the dose of dopaminergic drugs for better motor control.

Some of these cases show that PDP symptoms may occur at various intervals in the course of PD, and physicians should be aware of the time course of occurrence. Minor symptoms such as passage hallucinations are likely precursors to more bothersome hallucinations (formed visual and auditory hallucinations) and should be carefully monitored. PDP hallucinations include not only visual and auditory forms but also olfactory and tactile sensations, particularly at later phases of evolution [4].

In some of the cases, however, the patients' history did not reveal a pattern of progression of PDP symptoms as described previously [14, 41]. Indeed, the chronology of PDP progression is controversial [16, 27], based on divergent study data [16, 42]. A recent literature review of 184 case reports of delusions in PDP found that 50% were isolated from other symptoms [16]. In the cases where delusions were isolated rather than accompanied by other PDP symptoms, patients had an earlier onset of PD (46 years vs. 55 years) and lower rates of cognitive impairment (8.0% vs. 26.8%). This is consistent with some of the cases reported here.

Some patients in our case series did not tolerate the 34 mg pimavanserin dose. During the pivotal phase 3 clinical trial of pimavanserin, 10 (10.5%) patients in the pimavanserin group (*n* = 95) discontinued treatment because of AEs, including four who discontinued because of psychotic disorder or hallucination within 10 days of starting the treatment. The lower dose of pimavanserin was not tested during the trial, but in this case series, two patients were able to tolerate pimavanserin after the dose was reduced to 17 mg. In addition, administering pimavanserin in the evening successfully averted daytime sedation. It is worth noting that sedation was not among the most frequent AEs reported in the pivotal phase 3 trial of pimavanserin (i.e., AEs occurring in ≥5% of patients in either treatment group) [38]; however, a medical center chart review of patients who received pimavanserin reported that “somnolence” was the second most common AE, occurring in 3 patients (3%) [43]. Among the other notable features in this case series, a nonpharmacological approach was applied that included alleviating life stresses and improving environmental factors such as lighting to reduce the risk of hallucinations; however, this approach must be balanced against the potentially more effective modality of antipsychotic therapy.

In conclusion, this report details patient cases from the clinical setting that illustrate approaches and challenges that clinicians are using to manage PDP symptoms and the use of pimavanserin. These 11 cases demonstrate that pimavanserin was relatively well tolerated and improved the symptoms of PDP in patients at different stages of the disease with varying symptom severity. The cases also highlight the lack of clinical information, recommendations, and guidelines for using pimavanserin in PDP that occurs along with comorbidities (e.g., mild cognitive impairment, mood disorders, and sleep disturbances). Cholinesterase inhibitors may be useful in reducing psychoses in PD patients with dementia [33, 34].

There are no recommendations regarding the use of pimavanserin at doses lower or higher than 34 mg or in conjunction with other antipsychotic medications such as quetiapine. All of these concerns highlight the need for more studies designed to address practical issues with the use of pimavanserin for PDP.

## Figures and Tables

**Table 1:** Summary of 11 cases of Parkinson's disease psychosis.

Case #	Date of PD diagnosis	Date of initial visit	Age (y)	Sex	PD duration (y)^*∗*^	Medication history	Comorbid conditions	Primary complaint
1	2013	2016	79	M	5	Rasagiline	Depression	Tremor
						CD/LD		

2	2004	2016	71	M	14	Citalopram	Diabetes and hypertension	Generalized dyskinesias and tremor
						Quetiapine		

3	2011	2017	79	M	6	None	Hearing loss	Auditory hallucinations

4	2006	2018	71	M	12	CD/LD	Depression	Persistent false perceptions and daily hallucinations
						Rasagiline		
						Sertraline		

5	2006	2016	68	M	10	CD/LD	None noted	Difficulty walking; short-term memory loss
						Selegiline		

6	1999	NA	84	F	20	CD/LD	None noted	Persisting tremor and movement disorders
						Pramipexole		
						Amantadine		
						Rasagiline		

7	2016	2017	73	M	1^†^	CD/LD	Mild cognitive dysfunction	Symptoms consistent with RBD
						Duloxetine	Depression and anxiety	

8	NA	NA	68	M	9	Levodopa	None noted	Severe refractory tremors and motor fluctuations; evaluate for DBS

9	2002	2017	69	F	15	Levodopa	Depression and mild cognitive impairment	Tremors, bradykinesia, rigidity, and severe dyskinesia
						Amantadine		
						Rotigotine		

10	NA	NA	74	F	12	Levodopa	Depression and anxiety	Left upper-extremity tremor, decreased dexterity, and gait instability

11	NA	NA	76	M	4	None noted	Cognitive impairment	Shuffling gait, slowness, and drooling

^*∗*^At the time of evaluation. ^†^Possible PD symptoms had been present for 6 years. CD/LD, carbidopa/levodopa; DBS, deep brain stimulation; PD, Parkinson's disease; PDP, Parkinson's disease psychosis; RBD, rapid eye movement sleep behavior disorder.

**Table 2:** Treatment, disease course, and key takeaways from each case.

Case #	Treatment	Disease course	Takeaways
1	PIM 34 mg	Improved PDP symptoms at 4 weeks	(i) Delusions may occur isolated from other, typically earlier, symptoms of PDP such as illusions and hallucinations
2	PIM 17 mg; excess sedation with 34 mg	PDP symptoms resolved by week 5	(ii) PDP may be mistaken for dementia
			(iii) Pimavanserin dosed at 17 mg/d may be effective in some patients

3	PIM 34 mg reduced to 17 mg for sedation	At PIM 17 mg, PDP symptoms resolved by week 4	(i) Pimavanserin may be added without disrupting or adversely affecting other multidrug PD regimens
4	PIM 34 mg	Reduction in PDP symptoms at 3 weeks and complete resolution of PDP and RBD symptoms at 6 weeks	(ii) Screening for psychosis may reveal PDP symptoms that might otherwise go unreported without elicitation by the clinician
5	Selegiline PIM 34 mg	Hallucinations continued with selegiline, although motor fluctuations improved; PIM resolved hallucinations	(iii) After an initial onset of symptoms, PDP may often progress in frequency and severity to require urgent treatment
6	PIM 34 mg	No response to pramipexole; after starting PIM, hallucinations improved	

7	Escitalopram 10 mg PIM 34 mg Donepezil 10 mg	Hallucinations had resolved with 1-2 weeks of PIM; RBD symptoms persisted, so started clonazepam; DC escitalopram and added venlafaxine	(i) RBD, along with other parasomnias, is associated with visual hallucinations and cognitive impairment in patients with PD
			(ii) Concomitant RBD in a patient with PD reporting symptoms of possible psychosis requires assessment if the symptoms are related to RBD or to PDP

8	DBS initiated PIM 34 mg	Symptoms worsened after DBS; after starting PIM, symptoms improved	(i) Patients with PD who have DBS may be at increased risk of PDP
			(ii) Pimavanserin may be effective for treating PDP in patients with DBS
9	PIM 34 mg Quetiapine 150 mg	Discontinued amantadine and rotigotine; PDP partly resolved	(iii) Both pimavanserin and another antipsychotic may be necessary to manage PDP
10	Nonpharmacological; quetiapine 75–100 mg; PIM 34 mg	No response to nondrug intervention; marked improvement at 6 weeks	

11	PIM 34 mg	After 4–6 weeks, marked improvement; no AEs	(i) PDP may emerge soon after PD diagnosis (e.g., within 1 year)
			(ii) PDP symptoms may be unrecognized in patients living in a nursing home or assisted living facility by the facility staff

AEs, adverse events; DBS, deep brain stimulation; DC, discontinued; PD, Parkinson's disease; PDP, Parkinson's disease psychosis; PIM, pimavanserin; RBD, rapid eye movement sleep behavior disorder. All cases constitute an AAN Class IV level of evidence and U level of recommendation.

## Data Availability

Data sharing is not applicable to this article as no datasets were generated or analyzed for this case series.

## Abbreviations

AEs: Adverse events
CD/LD: Carbidopa/levodopa
DBS: Deep brain stimulation
PD: Parkinson's disease
PDP: Parkinson's disease psychosis
RBD: Rapid eye movement sleep behavior disorder.
